# Adenosquamous carcinoma of the lung: Comparative CT and pathological features versus adenocarcinoma and squamous cell carcinoma

**DOI:** 10.1371/journal.pone.0352454

**Published:** 2026-06-24

**Authors:** Qianyao Yuan, Dai Zhang, Rui Xu, Wenjun Yao, Hong Zhao

**Affiliations:** Department of Radiology, The Second Affiliated Hospital of Anhui Medical University, Hefei, China; Noorda College of Osteopathic Medicine, UNITED STATES OF AMERICA

## Abstract

**Objective:**

Adenosquamous carcinoma (ASC) of the lung, containing both adenocarcinoma (AC) and squamous cell carcinoma (SCC) components, is associated with aggressive behavior and poor prognosis. Due to overlapping imaging features with AC and SCC, its preoperative diagnosis remains challenging. This study aimed to compare CT and pathological characteristics among ASC, AC, and SCC; identify key differentiators.

**Methods:**

This retrospective study included 27 patients with pathologically confirmed ASC who underwent surgical resection from November 2018 to January 2025. Forty cases each of AC and SCC, matched for age, sex, and smoking history, were selected for comparison. Clinical, radiological, and pathological features were analyzed. Variables with statistical significance (P < 0.05) in univariate analysis were further evaluated using multivariate logistic regression. The discriminatory performance of the models was assessed using the area under the receiver operating characteristic curve (AUC).

**Results:**

Compared with SCC, ASC lesions were more peripheral and associated with higher distance ratios (DR), pleural retraction, spiculation, and lymph node metastasis (LNM). Compared with AC, ASC tumors were larger, more often solid, and exhibited lobulation, bronchial cutoff sign, and advanced staging. Among the evaluated analyses, the combined pathology–imaging approach showed the highest discriminatory performance, with AUCs of 0.909 (ASC vs SCC) and 0.900 (ASC vs AC). LNM and DR were more strongly associated with ASC relative to SCC, whereas larger tumor size, ill-defined margins, and advanced stage were more strongly associated with ASC relative to AC.

**Conclusion:**

Certain CT imaging features, especially DR and LNM, were associated with ASC and may provide useful information for preoperative radiological assessment and subtype stratification.

## 1 Introduction

ASC of the lung is a rare but highly aggressive non-small cell lung cancer (NSCLC) subtype, histologically defined by the coexistence of AC and SCC components, each comprising at least 10% of the tumor volume [1–3]. Although ASC accounts for only 0.4% to 4% of all lung cancer cases [4,5], it shows more aggressive clinical behavior and significantly worse prognosis than either AC or SCC [6–9]. A large SEER database study reported a 5-year overall survival rate of 64.5% for ASC, significantly lower than that of AC (76.7%) and SCC (69.1%) [6]. In addition, ASC more frequently presented with higher histological grade, larger tumor long-axis diameter, and an intermediate LNM rate between the two single histologic types. These findings suggest that ASC has greater invasive potential and may be less responsive to treatment.

Current research on ASC primarily addresses its pathological classification, molecular targets, and prognostic indicators [10–15]. Although several studies have examined the CT imaging characteristics of ASC, most have described ASC in isolation, lacking systematic comparisons with other histologic lung cancer subtypes [7,16–18]. As a result, the distinctive imaging characteristics that differentiate ASC from other subtypes remain poorly understood. According to the literature, AC typically appears as a peripheral lesion with part-solid or ground-glass nodules [19], whereas SCC more often occurs centrally, presents a solid lesion, and is frequently associated with necrosis [20]. As a mixed-type tumor, ASC displays a heterogeneous spectrum of imaging findings, often combining characteristics of both AC and SCC. Previous studies have primarily conducted intra-group comparisons within ASC—such as peripheral versus central patterns or AC-dominant versus SCC-dominant subtypes—whereas comprehensive comparative analyses between ASC and either AC or SCC remain scarce. Consequently, the radiologic differentiation and anatomical localization of ASC relative to other lung cancer subtypes remain insufficiently characterized.

In clinical practice, the definitive diagnosis of ASC still primarily relies on postoperative pathology. To further explore the radiological characteristics of ASC, the present study systematically compares the clinical and imaging characteristics of ASC, AC, and SCC, with the goal of identifying imaging features potentially associated with ASC. Multivariate logistic regression analysis was employed to assess the relative contributions of each feature and the performance of their combined application. By elucidating the differential features of ASC relative to AC and SCC, this study aims to inform radiological assessment and provide additional information for preoperative subtype evaluation.

## 2 Materials and methods

### 2.1 Case selection

This retrospective study was approved by the Institutional Review Board of The Second Affiliated Hospital of Anhui Medical University, with a waiver of written informed consent (Approval No.: SL-YX2025–089). The data were accessed for research purposes between June 2025 and August 2025. Patient identifiers were used only for case retrieval within the hospital information system, and all data were anonymized prior to analysis; the authors had no access to identifiable participant information during or after data analysis. Electronic medical records from November 2018 to January 2025 were reviewed to identify eligible patients who met the following inclusion criteria: (1) histopathological confirmation of ASC, SCC, or AC of the lung following surgical resection; and (2) completion of both chest CT and pathological examination during hospitalization. Exclusion criteria included pulmonary metastases, multiple pulmonary lesions with an undetermined primary origin, or incomplete clinical or CT data.

A total of 27 patients with ASC were ultimately included, comprising 21 males and 6 females, aged 45–87 years (median age: 62 years). According to the 8th edition of the TNM classification by the Union for International Cancer Control (UICC), the pathological stages were: IA (n = 4), IB (n = 4), IIA (n = 5), IIB (n = 4), IIIA (n = 8), IIIB (n = 1), and IVA (n = 1). Additionally, A total of 300 cases each of AC and SCC diagnosed during the same period were reviewed. From these, 40 AC and 40 SCC cases were selected by matching age, sex, and smoking history with the ASC group. Comparative analysis was conducted to evaluate the clinicopathological and CT imaging features among the three groups.

### 2.2 Review of clinical, pathological, and imaging information

Clinical and pathological variables included: age (years), sex (male/female), smoking history, pathological stage, LNM, vascular invasion, pleural invasion, perineural invasion, and bronchial wall invasion. CT imaging features assessed were as follows: distance ratio (DR), tumor-involved lobe (involvement of two or more lobes was categorized as “other”), tumor location, tumor long-axis diameter (maximum axial length of the lesion), lesion margin (well-defined or ill-defined), density (homogeneous or heterogeneous), tumor composition, lobulation, spiculation, air bronchogram, pleural retraction sign, bubble-like lucency, cavitation, calcification, peritumoral inflammatory response, bronchial cutoff, obstructive atelectasis, perilesional emphysema, and pleural effusion.

**Fig 1:**
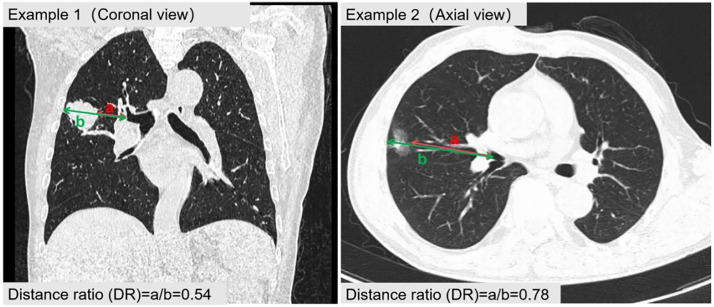
Illustration of DR measurement. Two distances are defined: (a) from the hilum to the proximal margin of the tumor; and (b) from the hilum, through the center of the tumor, to the visceral pleura. The DR is calculated as the ratio of (a) to (b).

DR was defined as the ratio of the distance from the hilum to the proximal margin of the tumor to the distance from the hilum through the center of the lesion to the visceral pleura [21], as illustrated in Fig 1. Tumor location was classified as central if the lesion originated at or above the segmental bronchus, and peripheral if located distal to the segmental bronchi. A well-defined margin was described as a smooth tumor border with clear demarcation from the surrounding lung parenchyma, without spiculation or ground-glass opacity. Homogeneous density referred to internal uniformity of the lesion without cystic changes, necrosis, calcification, or heterogeneous enhancement. Tumor composition was categorized as solid, part-solid, or pure ground-glass based on CT appearance. Solid lesions were defined as those with no visible ground-glass opacity that completely obscured the underlying lung architecture; part-solid lesions contained both solid and ground-glass components, with the solid portion partially obscuring the lung parenchyma; and pure ground-glass lesions appeared as hazy increased attenuation that did not obscure the underlying bronchial and vascular structures. Peritumoral inflammatory response was defined as the presence of surrounding ground-glass opacity, consolidation, or fibrous strands. A bubble-like lucency was defined as a round or oval radiolucent area within the lesion measuring less than 5 mm in diameter.

All CT imaging features were independently reviewed by two radiologists with at least 3 years of diagnostic experience. In cases of disagreement, consensus was reached through discussion.

### 2.3 Statistical analysis

All statistical analyses were performed using SPSS software version 27.0 (IBM Corporation, Armonk, NY, USA). A P value < 0.05 was considered statistically significant. Student’s t-test was used for continuous variables with a normal distribution, while the Mann–Whitney U test was applied to non-normally distributed continuous variables. Categorical variables were analyzed using the χ² test or Fisher’s exact test, as appropriate. Variables found to be statistically significant in univariate analysis were further tested for multicollinearity. Multicollinearity was evaluated by calculating the variance inflation factor (VIF); a VIF < 5 was considered acceptable. Significant variables were then included in multivariate logistic regression analysis to identify features independently associated with ASC. Based on variable types, pathology-based analyses, imaging-based analyses, and combined pathology–imaging analyses were performed. The discriminative performance of each analysis approach was evaluated using receiver operating characteristic (ROC) curve analysis, and the AUC was calculated.

## 3 Results

### 3.1 Clinical and pathological characteristics of ASC

Among the 27 patients with pulmonary ASC, the mean age was 68.48 ± 9.91 years. Males accounted for 77.8% (21/27) of the cohort, and 25.9% (7/27) had a history of smoking. LNM was observed in 48.1% (13/27) of cases, vascular invasion in 44.4% (12/27), pleural invasion in 29.6% (8/27), perineural invasion in 3.7% (1/27), and bronchial wall invasion in 7.4% (2/27).

### 3.2 Comparison of clinical, pathological, and imaging features among ASC, SCC, and AC

**Fig 2:**
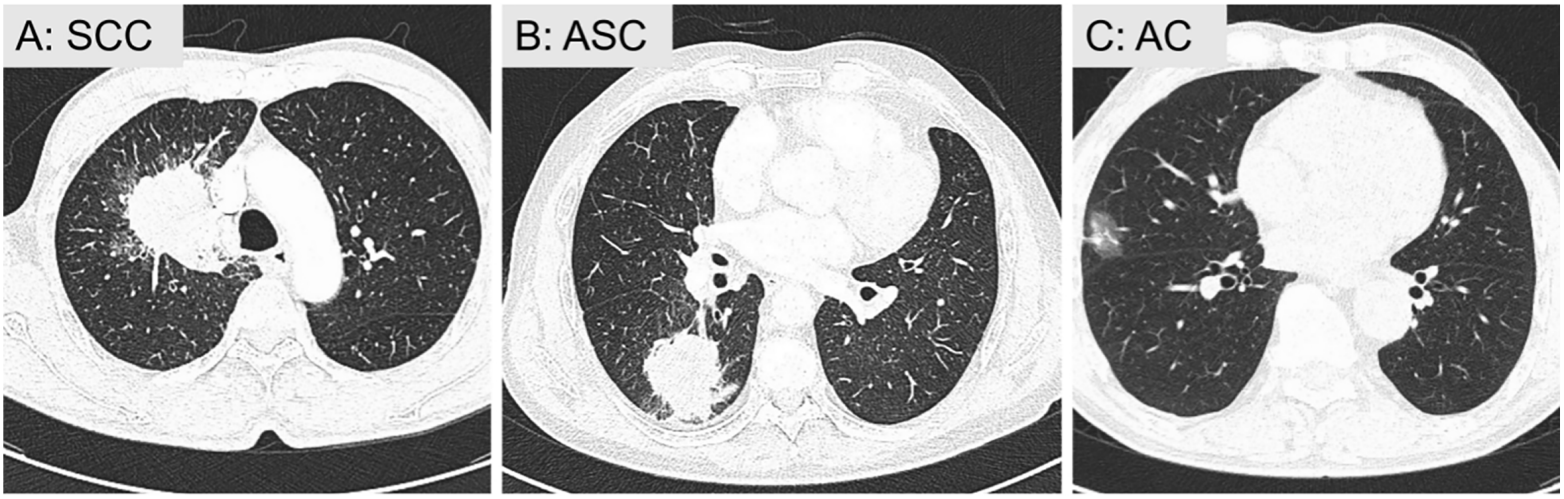
Representative CT images of three patients with different histological subtypes. **A.** A 68-year-old male with a DR of 0.00. Pathologically confirmed SCC without LNM. **B.** A 73-year-old male with a DR of 0.45. Pathologically confirmed ASC with LNM. **C.** A 60-year-old male with a DR of 0.76. Pathologically confirmed AC without LNM.

Forty cases each of SCC and AC were selected and matched with the 27 ASC cases by age, sex, and smoking history. Compared with SCC, ASC cases were more frequently peripheral in location and more commonly exhibited pleural retraction sign, spiculation, and LNM. In contrast, the incidence of bronchial wall invasion and perineural invasion was lower in ASC. The mean DR was significantly higher in ASC (0.53 ± 0.18) than in SCC (0.19 ± 0.25), P < 0.05. When compared with AC, ASC cases demonstrated a significantly larger tumor long-axis diameter and were more often solid in composition. Additionally, ASC was associated with higher frequencies of LNM, ill-defined margins, lobulation, peritumoral inflammatory response, bronchial cutoff sign, and advanced pathological stage (P < 0.05 for all comparisons). Detailed results are presented in Table 1 and Fig 2.

**Table 1:** Comparison of clinical, pathological, and imaging features between ASC and SCC.

Variable	ASC (n = 27)	AC (n = 40)	SCC (n = 40)	P value (AC versus ASC)	P value (SCC versus ASC)
Age (years)	68.5 ± 9.9	64.7 ± 9.6	67.0 ± 7.4	0.119	0.487
Sex				0.086	0.171
Male	21 (77.8%)	23 (57.5%)	37 (92.5%)		
Female	6 (22.2%)	17 (22.5%)	3 (7.5%)		
Smoking history	7 (25.9%)	6 (15.0%)	8 (20.0%)	0.267	0.568
Pathological stage				0.004	0.190
I	8 (29.6%)	28 (70.0%)	19 (47.5%)		
II	9 (33.3%)	4 (10.0%)	14 (35.5%)		
III	9 (33.3%)	8 (20.0%)	7 (17.5%)		
IV	1 (3.7%)	0 (0.0%)	0 (0.0%)		
Lymph node metastasis	13 (48.1%)	9 (22.5%)	9 (22.5%)	0.028	0.028
Vascular invasion	12 (44.4%)	11 (27.5%)	16 (40.0%)	0.152	0.780
Pleural invasion	8 (29.6%)	13 (32.5%)	13 (32.5%)	0.804	0.804
Perineural invasion	1 (3.7%)	5 (12.5%)	13 (32.5%)	0.423	0.004
Bronchial wall invasion	2 (7.4%)	6 (15.0%)	16 (40.0%)	0.578	0.030
Tumor long-axis diameter (mm)	38.3 ± 12.2	28.3 ± 11.7	39.7 ± 20.7	0.01	0.750
Distance ratio (DR)	0.53 ± 0.18	0.48 ± 0.23	0.19 ± 0.25	0.41	<0.010
Tumor location				0.675	0.010
Central	8 (29.6%)	10 (25.0%)	28 (70.0%)		
Peripheral	19 (70.4%)	30 (75.0%)	12 (30.0%)		
Tumor-involved lobe				0.440	0.707
RUL	7 (25.9%)	8 (20.0%)	13 (32.5%)		
RML	0 (0.0%)	4 (10.0%)	0 (0.0%)		
RLL	8 (29.6%)	8 (20.0%)	8 (20.0%)		
LUL	6 (22.2%)	9 (22.5%)	9 (22.5%)		
LLL	6 (22.2%)	11 (27.5%)	8 (20.0%)		
Others	0 (0.0%)	0 (0.0%)	2 (5.0%)		
Lesion margin				<0.001	0.142
Well-defined	5 (18.5%)	24 (60.0%)	14 (35.0%)		
Ill-defined	22 (81.5%)	16 (40.0%)	26 (65.0%)		
Density				0.451	0.648
Homogeneous	14 (51.9%)	17 (42.5%)	23 (57.5%)		
Heterogeneous	13 (48.1%)	23 (57.5%)	17 (42.5%)		
Tumor composition				0.002	0.816
Solid	25 (92.6%)	22 (55.0%)	34 (85.0%)		
Part-solid	2 (7.4%)	10 (25.0%)	6 (12.5%)		
Pure ground-glass	0 (0.0%)	8 (20.0%)	1 (2.5%)		
Lobulation	22 (81.5%)	18 (45.0%)	20 (50.0%)	0.003	0.090
Spiculation	16 (59.3%)	15 (38.5%)	9 (22.5%)	0.096	0.020
Air bronchogram	5 (18.5%)	15 (37.5%)	2 (5.0%)	0.096	0.172
Pleural retraction	18 (66.7%)	20 (50.0%)	10 (25.0%)	0.177	0.010
Bubble-like lucency	4 (14.8%)	6 (15.0%)	4 (10.0%)	1.00	0.832
Cavitation	2 (7.4%)	2 (5.0%)	3 (7.5%)	1.00	1.000
Calcification	2 (7.4%)	1 (2.5%)	8 (20.0%)	0.726	0.285
Peritumoral inflammatory response	18 (66.7%)	14 (35.0%)	32 (80.0%)	0.011	0.219
Bronchial cutoff	16 (59.3%)	11 (27.5%)	32 (80.0%)	0.009	0.065
Obstructive atelectasis	2 (7.4%)	0 (0.0%)	8 (20.0%)	0.310	0.285
Perilesional emphysema	9 (33.3%)	6 (15.0%)	10 (25.0%)	0.077	0.458
Pleural effusion	4 (14.8%)	2 (5.0%)	7 (17.5%)	0.345	0.771

### 3.3 Multivariate analysis of differential indicators between ASC and SCC

**Fig 3:**
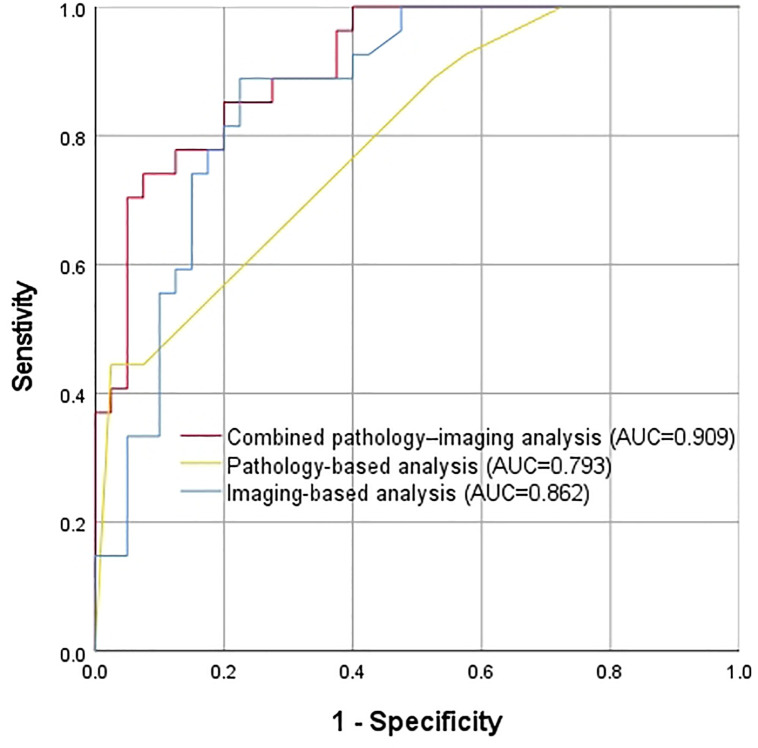
ROC curves for the combined pathology–imaging analysis, imaging-based analysis, and pathology-based analysis in ASC versus SCC.

To comprehensively evaluate the differential features associated with ASC relative to SCC, variables with statistical significance (P < 0.05) in univariate analysis were included in a multivariate logistic regression model. Collinearity diagnostics showed that all included variables had variance inflation factor (VIF) values < 5, indicating no significant multicollinearity. Taking SCC as the reference group, the combined pathology–imaging analysis identified LNM (OR = 8.498, 95% CI: 1.442–50.069, P = 0.018) and DR (OR = 167.915, 95% CI: 1.721–16379.264, P = 0.028) as features independently associated with ASC (Table 2). To further compare the discriminative power of different variable types, separate pathology-based, imaging-based, and combined pathology–imaging analyses were performed. Within this study cohort, the combined pathology–imaging analysis showed the highest discriminatory performance, with an AUC of 0.909 (95% CI: 0.842–0.977). The corresponding AUC values were 0.793 for the pathology-based analysis and 0.862 for the imaging-based analysis, as shown in Fig 3.

**Table 2:** Multivariate logistic regression analysis of features associated with ASC using SCC as reference.

Variable	Combined Pathology–Imaging Analysis		Imaging-Based Analysis		Pathology-Based Analysis	
	OR (95%CI)	P value	OR (95%CI)	P value	OR (95%CI)	P value
Lymph node metastasis	8.498 (1.442–50.069)	0.018			7.943 (1.773–35.578)	0.007
Perineural invasion	0.057 (0.003–1.175)	0.063			0.049 (0.004–0.560)	0.015
Bronchial wall invasion	0.263 (0.034–2.065)	0.204			0.152 (0.026–0.908)	0.039
Distance ratio (DR)	167.915 (1.721–16,379.264)	0.028	501.095 (9.868–25,445.825)	0.002		
Tumor location	2.842 (0.294–27.437)	0.367	3.251 (0.476–22.220)	0.229		
Spiculation	3.405 (0.753–15.394)	0.111	2.772 (0.690–11.130)	0.151		
Pleural retraction	1.747 (0.341–8.942)	0.503	1.812 (0.445–7.379)	0.406		

### 3.4 Multivariate analysis of differential indicators between ASC and AC

**Fig 4:**
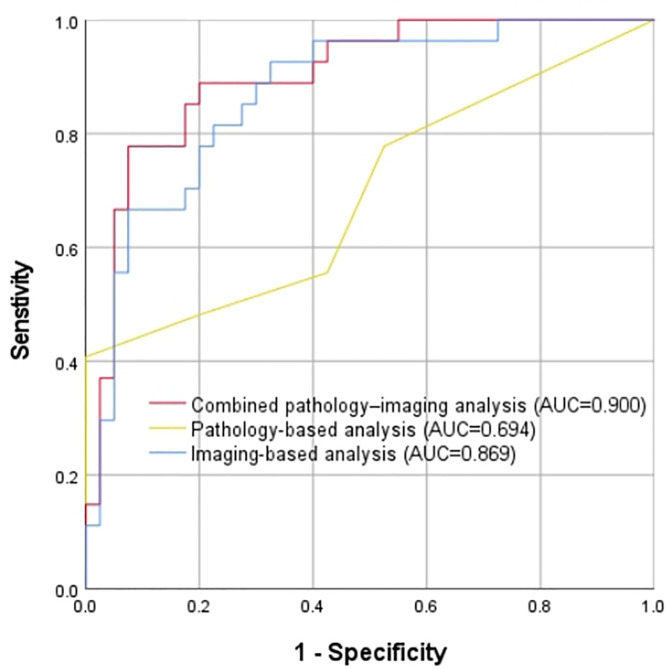
ROC curves for the combined pathology–imaging analysis, imaging-based analysis, and pathology-based analysis in ASC versus AC.

To evaluate the differential features associated with ASC relative to AC, multivariate logistic regression analysis was conducted following the same methodology as described above. Collinearity diagnostics showed that all included variables had variance inflation factor (VIF) values < 5, indicating no significant multicollinearity. Using AC as the reference group, the combined pathology–imaging analysis identified tumor long-axis diameter (OR = 1.094, 95% CI: 1.007–1.188, P = 0.033), lesion margin (OR = 6.623, 95% CI: 1.282–34.214, P = 0.024), and pathological stage (OR = 2.692, 95% CI: 1.094–6.626, P = 0.031) as features independently associated with ASC (Table 3). To further compare the discriminative power of different variable types, separate pathology-based, imaging-based, and combined pathology–imaging analyses were performed. Within this study cohort, the combined pathology–imaging analysis showed the highest discriminatory performance, with an area under the curve (AUC) of 0.900 (95% CI: 0.825–0.975). The corresponding AUC values were 0.694 for the pathology-based analysis and 0.869 for the imaging-based analysis, as shown in Fig 4.

**Table 3:** Multivariate logistic regression analysis of features associated with ASC using AC as reference.

Variable	Combined Pathology–Imaging Analysis		Imaging-Based Analysis		Pathology-Based Analysis	
	OR (95%CI)	P value	OR (95%CI)	P value	OR (95%CI)	P value
Pathological stage	2.692 (1.094–6.626)	0.031			3.034 (0.986–9.336)	0.053
Lymph node metastasis	1.069 (0.208–5.494)	0.936			2.196 (1.186–4.067)	0.012
Tumor long-axis diameter (mm)	1.094 (1.007–1.188)	0.033	1.091 (1.013–1.175)	0.021		
Lesion margin	6.623 (1.282–34.214)	0.024	7.854 (1.622–38.039)	0.010		
Lobulation	2.407 (0.424–13.668)	0.321	2.325 (0.487–11.099)	0.290		
Peritumoral inflammatory response	0.817 (0.134–4.999)	0.827	0.550 (0.104–2.916)	0.482		
Bronchial cutoff	0.460 (0.083–2.546)	0.374	0.409 (0.082–2.041)	0.276		
Tumor composition	5.867 (0.934–36.872)	0.059	9.774 (1.768–54.048)	0.009		

## 4 Discussion

Pulmonary ASC is a rare yet highly aggressive subtype of NSCLC, representing approximately 0.4–4% of all lung cancer cases [4,5]. Postoperative survival rates for ASC are significantly lower than those for AC and SCC, reflecting its more unfavorable prognosis and higher invasiveness [6–9,15]. Compared to tumors with a single histological subtype, ASC more frequently exhibits vascular invasion, pleural involvement, and distant metastasis. However, current research has focused mainly on ASC's survival outcomes and imaging features in isolation, with few comparative analyses against other histologic subtypes such as AC and SCC.

This study compared the imaging features of ASC with those of AC and SCC. ASC was more often located in the peripheral lung (70.4%), a distribution pattern similar to that of AC. However, with respect to tumor long-axis diameter, predominance of solid lesions, and peritumoral inflammatory response, ASC more closely resembled SCC. These findings indicate that ASC exhibits a hybrid imaging pattern, incorporating features of both AC and SCC. This hybrid pattern aligns with the clinical heterogeneity previously described by Wu et al [22]. In this cohort, 77.8% of patients with ASC were male, with a median age of 67 years—findings consistent with prior reports that ASC predominantly affects older male individuals [23,24].

To further explore the origin of ASC imaging features, this study compared specific imaging features between ASC and SCC. ASC showed significantly higher frequencies of pleural retraction (66.7% vs. 25%, P = 0.01), spiculation (59.3% vs. 22.5%, P = 0.02), and LNM (48.1% vs. 22.5%, P = 0.028), whereas bronchial wall invasion (7.4% vs. 40%, P = 0.03) and perineural invasion (3.7% vs. 32.5%, P = 0.004) were significantly less frequent compared to SCC. These differences may be attributed to the adenocarcinomatous component of ASC, which contributes to its peripheral distribution and pleural traction features commonly seen in AC. Lung adenocarcinomas often originate from small bronchi or alveolar type II epithelial cells [25,26], and typically present as peripheral lesions with pleural retraction and spiculation [27,28]. Moreover, within ASC, the adenocarcinoma component exhibits a greater propensity for lymph node metastasis. [7,29,30], consistent with our finding that the rate of LNM in ASC was significantly higher than in SCC. Multivariate analysis further supported this finding, identifying LNM as a factor independently associated with ASC compared with SCC (OR = 8.498, 95% CI: 1.442–50.069, P = 0.018).

ASC also exhibits distinct imaging features compared to AC, in addition to those differentiating it from SCC. ASC more frequently demonstrates a solid-predominant lesion pattern (92.6% vs. 55%), ill-defined margins (81.5% vs. 40%), and peritumoral inflammatory response (66.7% vs. 35%), suggesting greater invasiveness. Notably, lobulation (81.5% vs. 45%, P = 0.003) and bronchial cutoff sign (59.3% vs. 27.5%, P = 0.009) occur significantly more often in ASC than in AC, which may indicate a more infiltrative growth pattern. The LNM rate is also higher in ASC (48.1% vs. 22.5%), consistent with previous studies [23,29], supporting its aggressive biological behavior. Furthermore, the mean tumor long-axis diameter in ASC (38.3 ± 12.2 mm) is significantly larger than that in AC (28.3 ± 11.7 mm, P = 0.01), though slightly smaller than in SCC (39.7 ± 20.7 mm). These findings suggest that ASC may exhibit intermediate biological characteristics between AC and SCC, presenting a “hybrid” or “intermediate-type” profile.

The definition of tumor location varies slightly among different researchers [31–34].To objectively quantify tumor location and peripheral distribution, this study introduces the DR, defined as the ratio of the distance from the hilum to the proximal tumor margin to the distance from the hilum through the tumor center to the pleura. This metric aims to reduce the subjectivity inherent in the traditional classification of central vs. peripheral tumors. The DR for ASC (0.53 ± 0.18) was significantly higher than that for SCC (0.19 ± 0.25, P < 0.01), indicating a stronger tendency for peripheral growth in ASC. This finding aligns with the higher prevalence of pleural retraction in ASC and may serve as a useful reference for differentiating ASC from SCC. In multivariate analysis, DR was also identified as a factor independently associated with ASC (OR = 167.915, 95% CI: 1.721–16,379.264, P = 0.028), However, the extremely wide confidence interval suggests substantial statistical uncertainty, likely related to the limited sample size, and therefore this finding should be interpreted with caution.

Based on the analysis of the above differential indicators, this study performed pathology-based, imaging-based, and combined analyses to evaluate the relative contributions of different features associated with ASC. Within this study cohort, the combined pathology–imaging analysis showed the highest discriminatory performance, with an AUC of 0.909 for ASC versus SCC and 0.900 for ASC versus AC. Both values were higher than those observed in the pathology-based or imaging-based analyses. These findings suggest that integrating multidimensional information may better characterize the features associated with ASC within this study cohort. It is important to note that the models developed in this study are exploratory and primarily intended for hypothesis generation. Some included variables were derived from postoperative pathological data and were primarily incorporated for exploratory analysis and hypothesis generation, rather than for direct preoperative clinical application.

This study has several limitations. (1) It is a retrospective, single-center study with a relatively small sample size for ASC (n = 27), which may limit the robustness and generalizability of the findings. In addition, the relatively low smoking prevalence in this Asian cohort may differ substantially from that reported in Western NSCLC populations, potentially reflecting differences in smoking exposure, genetic background, and tumor biology. Therefore, the generalizability of these findings to other populations should be interpreted with caution. (2) Some variables were derived from postoperative pathological results and primarily reflect characteristics associated with ASC. Therefore, they cannot be directly applied to preoperative clinical decision-making. (3) Imaging features were not integrated with molecular data (e.g., EGFR, KRAS mutations), limiting the ability to evaluate potential correlations between imaging phenotypes and genetic profiles. (4) The study did not analyze whether the proportion of adenocarcinomatous versus squamous components (AC-dominant vs. SCC-dominant) within ASC influences imaging features. Future research should focus on large-scale, multicenter studies incorporating radiomics and molecular profiling to validate and expand the current findings.

## 5 Conclusion

Several CT imaging features, particularly DR and LNM, were associated with ASC and may provide useful information for preoperative radiological assessment and subtype stratification. In addition, larger tumor size, ill-defined margins, and advanced pathological stage were more strongly associated with ASC relative to AC. Given the limited sample size and the inclusion of postoperative pathological variables, these findings should be interpreted with caution. Further validation in large-scale, multicenter studies is warranted.
